# Improved Estimates of Population Exposure in Low-Elevation Coastal Zones of China

**DOI:** 10.3390/ijerph16204012

**Published:** 2019-10-19

**Authors:** Xuchao Yang, Chenming Yao, Qian Chen, Tingting Ye, Cheng Jin

**Affiliations:** Ocean College, Zhejiang University, Zhoushan 310027, China; 3150100216@zju.edu.cn (C.Y.); chenqian824@zju.edu.cn (Q.C.); tingting.ye@zju.edu.cn (T.Y.); jincheng95@zju.edu.cn (C.J.)

**Keywords:** LECZ, population exposure, random forest, Cubist, point-of-interest

## Abstract

With sea level predicted to rise and the frequency and intensity of coastal flooding expected to increase due to climate change, high-resolution gridded population datasets have been extensively used to estimate the size of vulnerable populations in low-elevation coastal zones (LECZ). China is the most populous country, and populations in its LECZ grew rapidly due to urbanization and remarkable economic growth in coastal areas. In assessing the potential impacts of coastal hazards, the spatial distribution of population exposure in China’s LECZ should be examined. In this study, we propose a combination of multisource remote sensing images, point-of-interest data, and machine learning methods to improve the performance of population disaggregation in coastal China. The resulting population grid map of coastal China for the reference year 2010, with a spatial resolution of 100 × 100 m, is presented and validated. Then, we analyze the distribution of population in LECZ by overlaying the new gridded population data and LECZ footprints. Results showed that the total population exposed in China’s LECZ in 2010 was 158.2 million (random forest prediction) and 160.6 million (Cubist prediction), which account for 12.17% and 12.36% of the national population, respectively. This study also showed the considerable potential in combining geospatial big data for high-resolution population estimation.

## 1. Introduction

Coastal areas are associated with large and growing concentrations of human population and socioeconomic activities, including many large cities of the world [1]. Although a coastal location provides many benefits, it also exposes people and assets to a variety of natural and climate change-related hazards, such as typhoon, storm surge, and sea level rise [2], especially in low-elevation coastal zones (LECZ). The LECZ is defined as a contiguous area along the coast that is <10 m above sea level [3]. According to estimates from the Global Rural Urban Mapping Project (GRUMP) gridded population dataset for 2000, this zone covers 2% (2.7 million km^2^) of the world’s land area but contains 10% (634 million) of the world’s total population [3]. Following studies have quantified the size of populations residing in LECZ by utilizing two commonly used global population datasets, that is, LandScan and GRUMP [4,5]. According to the LandScan population dataset (https://landscan.ornl.gov/), 690 million people in 2006 [4] and 726 million in 2008 lived in the global LECZ [5]. These studies showed that inherent uncertainties of the input datasets and methods will likely affect conclusions, and variations in results were highly dependent on the input datasets [4,5]. All previous studies were heavily reliant on the accuracy of the gridded population dataset and the digital elevation model (DEM) data. Most studies used gridded population datasets with a spatial resolution of 1 × 1 km, which captures more area than finer resolution, thereby overestimating the LECZ land area and population relative to finer grid [6]. Therefore, high-resolution population datasets are needed to understand the populations at risk in LECZ.

With 18,000 km of coastlines and 14,000 km of island shorelines, China has a huge LECZ. China also has the largest population in LECZ, with over 126 million people in 2000 [3]. Since 1978, the Chinese government has launched the reform and openness policy, with a shift of economic development focus from inland to coastal areas. As a result, coastal areas have experienced rapid economic growth and urbanization. The most populous and economically developed cities, such as Shanghai, Guangzhou, Shenzhen, and Tianjin, are all located in the LECZ of China and attract numerous migrants from inland China [7]. According to the LandScan population dataset, Mondal and Tatem [5] suggested the LECZ population of over 173 million in 2008, whereas Liu, Wen [8] reported a lower population than the data above of 165 million in 2011. The results of these two studies seem to be counterintuitive because China is rapidly urbanizing, particularly along the coastal zones. The urban populations in China’s LECZ grow particularly rapidly [3]. Therefore, the real size of human populations in the LECZ of China and how they are distributed should be assessed. Previous attempts to determine the actual populations have been undertaken by using population datasets with coarse resolutions [3,5,8]. With the increase in the availability of geospatial big data that are highly correlated with human activities, detailed estimations of coastal population exposure are possible. For example, previous studies showed the considerable potential of point-of-interest (POI) [9,10] and Sina Weibo check-in data [11] in high-resolution population mapping.

In this study, we aim to quantify the magnitude and spatial distribution of population in China’s LECZ. Two machine learning methods that utilize multiple satellite images and POI data were adopted to disaggregate census population data in coastal provinces and municipalities of China to 100 × 100 m grids. Compared with WorldPop data, our methods can generate high-resolution population grid maps with higher accuracy. Then, the spatial distribution of LECZ in China and its population exposure were estimated by combining the resulting coastal population dataset and digital elevation model.

## 2. Data and Methods

**Figure 1:**
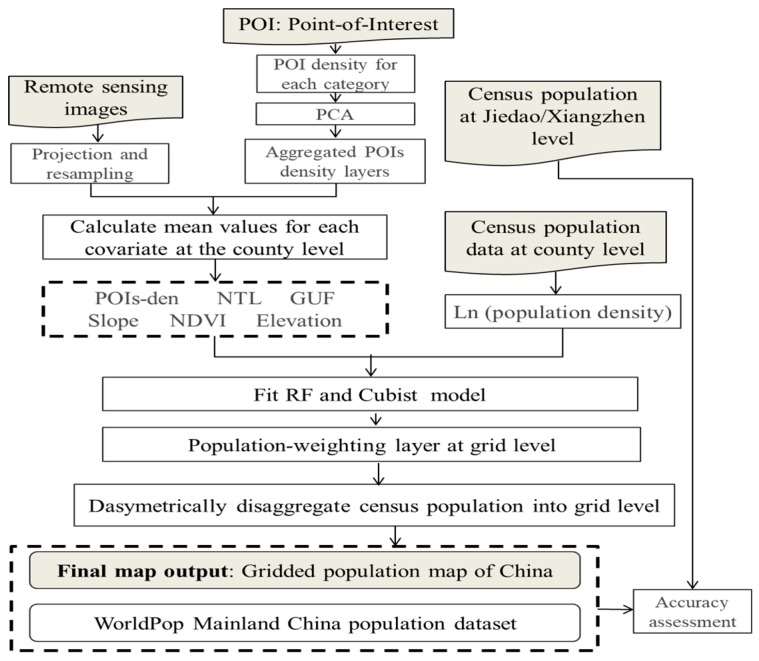
Flowchart of the production of population maps in coastal China. (PCA: Principal Component Analysis, NTL: Nighttime Light, GUF: Global Urban Footprint, NDVI: Normalized Difference Vegetation Index, RF: Random Forest).

Table 1 lists the datasets in this research, including population census data, nighttime light, vegetation index, POIs, and other ancillary data. Figure 1 outlines the general process used for data preparation, modeling, and validation, as follows:

### 2.1. Population Census Data and Administrative Boundaries

Population census counts for 2010 in coastal provinces/municipalities of China were obtained from the Sixth National Population Census of Mainland China (excluding Hong Kong, Macao, and Taiwan) and matched to administrative boundaries at the county level (administrative level 3; 978 units) and Jiedao/Xiangzhen level (administrative level 4; 13,065 units), respectively. The county-level census data were used in the actual model implementation, whereas the Jiedao/Xiangzhen-level data were held in reserve for model accuracy assessment.

### 2.2. Remote Sensing Data and Preprocessing

The global radiance-calibrated nighttime lights (NTL) product for 2010 was downloaded from the National Oceanic and Atmospheric Administration National Geophysical Data Center (F16_20100111-20101209_rad_v4, https://ngdc.noaa.gov/eog/dmsp/download_radcal.html). This product, with a spatial resolution of 30 arc second (~1 km), solves the saturation problem that occurs in the widely used stable light image composites of the Defense Meteorological Satellite Program’s Operational Linescan System (DMSP-OLS) product [12].

The normalized difference vegetation index (NDVI) data in 2010 were derived from the vegetation sensor on board the Satellite Pour l’Observation de la Terre (SPOT) program and downloaded from the Vlaamse Instelling Voor Technologisch Onderzoek (VITO, http://www.vgt.vito.be/). The SPOT S10 NDVI (Vlaamse Instelling Voor Technologish Onderzoek, Belgium) data exhibited a spatial resolution of 1 km^2^ and a temporal frequency of 10 days. A maximum value composite method was used to generate annual maximum NDVI images to separate human settlements from bare soils and remove the effect of cloud contamination in such a large study area.

NDVI_max_ = MAX (NDVI_1_, NDVI_2_,…, NDVI_36_) (1)

where NDVI_1_, NDVI_2_, …, NDVI_36_ are the 36 × 10 day SPOT NDVI images in 2010.

The NTL imagery and the NDVI_max_ image with geographic (Lat/Lon) projection were resampled to 100 m and then reprojected to an Albers Conical Equal Area projection by using the nearest neighbor algorithm.

The original DEM data used in the present study comprised the ASTER GDEM Version 2 (NASA Jet Propulsion Lab, the United States, and Japan’s Ministry of Economy, Trade, and Industry, Japan), with a 30 m resolution, downloaded from the website of the Earth Remote Sensing Data Analysis Center of Japan (http://www.gdem.aster.ersdac.or.jp/search.jsp). The 30 m DEM data were resampled by using bilinear interpolation to generate a new dataset with a pixel size of 100 m. We included elevation and its derived slope.

The Global Urban Footprint (GUF) data is an open-access dataset that provides global spatial information about human existence on Earth [13]. This dataset not only shows details of the presence of population and infrastructures in large cities, but also contains the information of small settlements. The original ~84 m GUF data were resampled to generate a new dataset with a pixel size of 100 m.

The WorldPop China Mainland dataset was produced by using a random forest (RF) method. This method integrates satellite remote sensing data, such as land use and road networks, and provides the most detailed population distribution forecast map in mainland China, with a spatial resolution of 100 m. We compared the produced population maps of this study with the WorldPop dataset to evaluate their accuracy. This dataset product is available in the WorldPop project website (https://www.worldpop.org/).

### 2.3. POIs and Processing

POIs were provided by Baidu Map Services (http://map.baidu.com), which is the most widely used and the largest web map service provider in China. With the help of the application programming interfaces that were provided by Baidu, we fetched 2,577,524 POIs records in mainland China [9]. In Baidu Map Service, the definition of POI is given in the form of a semantic Chinese phrase, which does not need word segmentation or reclassification in advance [14]. In the Baidu POI dataset, 20 labels belong to the top-level category, including educational facilities (e.g., kindergartens, primary schools, middle schools, colleges, and universities), clinical facilities (e.g., general hospital, special hospital, and children’s hospital), retail stores, catering and entertainment services, and public service facilities (e.g., public toilet, telecom, and nursing services; Table 2). The planar kernel density estimation tool in ArcGIS was used to produce a smooth and continuous density surface of POIs for each category. We tested the different bandwidths from 500 m to 8000 m at the intervals of 100 m before determining the value of 2000 m [9]. To reduce the number of variable inputs in the model and the computational burden of the model, we used principal component analysis (PCA) to combine 20 POI density layers. The PCA method reforms a set of linearly related indicators into a new set of linearly independent variables by linearly transforming the coordinates of the original data space, thereby reducing dimensionality. According to the PCA results, the first principal component had the largest variance and contained nearly 90% of the information. Therefore, we only selected the first principal component and output it in the form of raster as a composite density surface (POIs-den) with a resolution of 100 × 100 m.

### 2.4. Cubist and Random Forest Regression

In this study, two classic and popular rule-based machine learning methods, including Cubist regression tree and RF, were chosen to model the relationships between population density and geographic variables. Both regression methods can use discrete and continuous variables as input variables [15]. Cubist is a commercial rule-based multivariate regression model [16,17] that produces multiple linear regression models in the terminal nodes of trees on the basis of M5 theory. The analytical results of the Cubist models consist of a set of rules, each of which rule has a related multivariate linear model [18]. Cubist creates an explicit model and gives relative importance on input predictors [19], thereby allowing an easy model interpretation. Cubist also has a much shorter run time than classification and regression tree methods [20].

The RF algorithm, as a nonparametric, nonlinear, and ensemble machine learning method, is characterized by a flexible and robust framework that allows disparate data types to interact with each other in the modeling process [21]. Compared with other ensemble methods, RF algorithm is robust to outliers, noise, and overfitting, and requires little in parameter specifications [21,22]. The RF model has an internal cross-validation component that estimates the prediction error of the model, thereby removing the need for a set-aside test set. During the modeling process of the RF, at each node of each tree, one-third of the data is held in reserve from the iterative bagging process and used to generate an out-of-bag (OOB) error, which provides an unbiased estimate of prediction error [23]. The prediction error of the entire RF model can be calculated by averaging the OOB error of all trees. The OOB error can be also used to evaluate the importance of each covariate by calculating the average percent increase in the mean squared error (MSE). Additional details on RF are found in the references [21,22].

### 2.5. Model Fitting and Dasymetric Population Mapping

The six raster layers of NTL, NDVI, elevation, slope, GUF, and POI density were aggregated by county level as independent variables, and the natural logarithm of the census population density was considered a dependent variable. Then, the relationships between geographic indicators and target population density were established using RF and Cubist models, respectively. The same raster layers were input to the fitted RF and Cubist models, and then the prediction layer was calculated. According to the dasymetric mapping method [9,24,25], the prediction layers of the two machine learning methods were considered as the distribution weight at pixel level, and census county-level population data were disaggregated into a 100 m spatial resolution grid, as follows:

(2) $$POP_{grid}=\frac{POP_{county}\times W_{grid}}{W_{county}}$$

where *W_grid_* is the population distribution weight for a 100 × 100 m gridded area, *W_county_* indicates the summed population distribution weight of a county, *POP_county_* represents the county’s census population, and *POP_grid_* is the distributed population for the gridded area.

### 2.6. Extracting Extent of China’s LECZ

**Figure 2:**
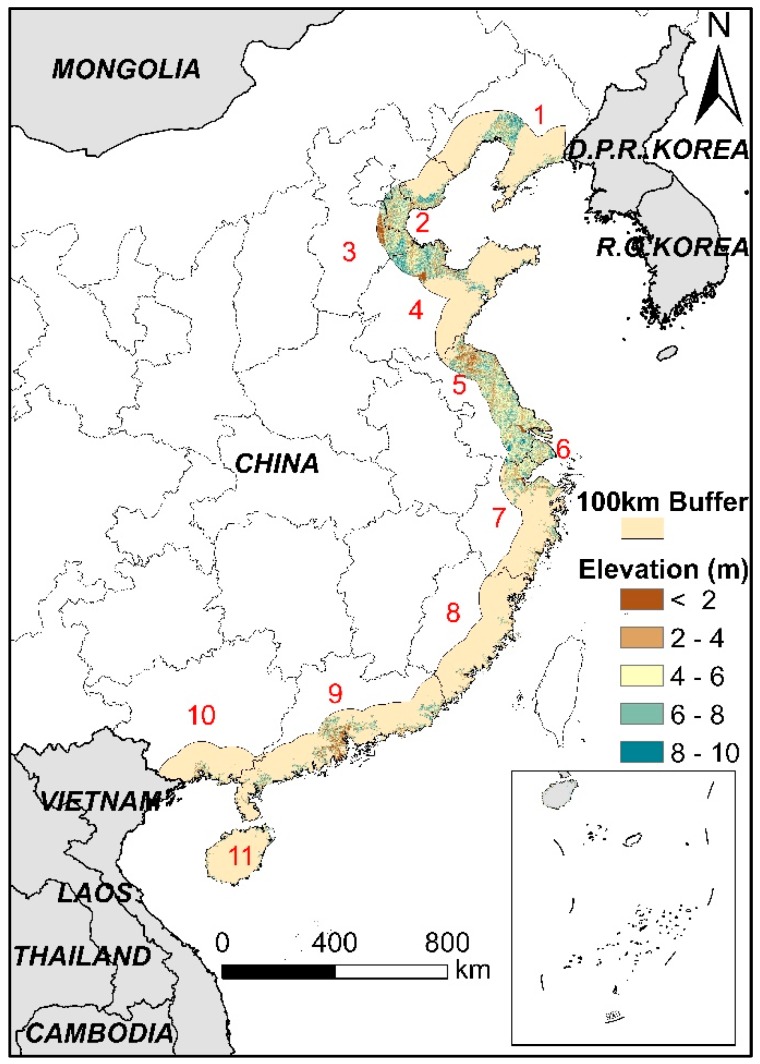
Distribution of China’s low-elevation coastal zones (LECZ). Numbers 1~11 represent Liaoning, Tianjin, Hebei, Shandong, Jiangsu, Shanghai, Zhejiang, Fujian, Guangdong, Guangxi, Hainan, respectively.

According to the definition of LECZ, that is, a coastal continuous zone with an elevation of <10 m and <100 km from the coastline [3], the extent of China’s LECZ was extracted using ArcGIS 10.2 software. The process is as follows: (1) a buffer area of 100 km from the coastline of China was output using a buffer tool, (2) all areas with an elevation of <10 m were extracted based on DEM raster, and (3) the two layers produced by steps 1 and 2 were overlaid to obtain the LECZ in China (Figure 2).

## 3. Results and Discussion

### 3.1. Accuracy Assessment of Population Mapping

To evaluate the accuracy of the prediction population maps from RF and Cubist models and compare the performance of two models, we collected the census population data at the Jiedao/Xiangzhen level (administrative level 4) from the study area. As a published, accurate gridded population dataset for China, the WorldPop dataset was also summed at the Jiedao/Xiangzhen level to compare the accuracy of the three population datasets. We selected the measures of mean relative error (MRE), mean absolute deviation (MAE), and root MSE (RMSE) to compare and analyze the errors of the above population dataset. Table 3 shows the results of the accuracy assessment for the population datasets predicted by RF, Cubist, and WorldPop. The MRE, MAE, and RMSE for population maps predicted by RF and Cubist were smaller than those of WorldPop. Therefore, the overall accuracy of the population maps obtained by the RF and Cubist models are higher than that of WorldPop at the Jiedao/Xiangzhen level. Specifically, the estimated population dataset from RF had better accuracy than that from Cubist, as demonstrated by the small MAE and RMSE values.

**Figure 3:**
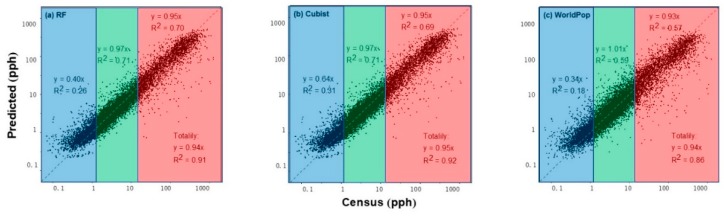
Scatterplots of census and predicted population densities by (a) RF, (b) Cubist, and (c) WorldPop at the township level. A log_10_–log_10_ transformation was conducted for population density. Red points represent townships with the top 20% of population densities among all samples, blue points indicate the townships with the smallest 20% population densities, and green points represent the remaining townships. pph: population per ha.

Figure 3 shows the relationship between the predicted population density and census population density. Each data point in the plots corresponded to a township, with 13,009 samples in total. The distribution census counts suggested an extremely good fit at medium population densities for the three datasets, with increasing errors at extremely low and high population densities (Figure 3). At extremely high population density (top 20%, red points), the underestimation in population estimation was significant, whereas overestimation was observed at extremely low population density (20%, blue points). This type of error showed that the dasymetric modeling process did not concentrate people heavily enough in high-population-density areas; instead, it spread estimations out to low-population-density areas. This problem was inherent to the population redistribution process in dasymmetric mapping literature [25,26]. However, by incorporating POI data, the predicted population data from RF (*R*^2^ = 0.91) and Cubist (*R*^2^ = 0.92) had higher overall accuracy and were closer to the one-to-one line than those from WorldPop (*R*^2^ = 0.86). The overestimations in townships with small populations and the underestimations in those with large populations in the WorldPop (*R*^2^ = 0.57 or *R*^2^ = 0.18) dataset were considerably alleviated in the predictions by integrating POI data in RF (*R*^2^ = 0.70 or *R*^2^ = 0.26) and Cubist (*R*^2^ = 0.69 or *R*^2^ = 0.31) models. The accuracy of our population datasets in medially populated townships also showed better performance than that of WorldPop (Figure 3).

### 3.2. Comparison between RF and Cubist Models

**Figure 4:**
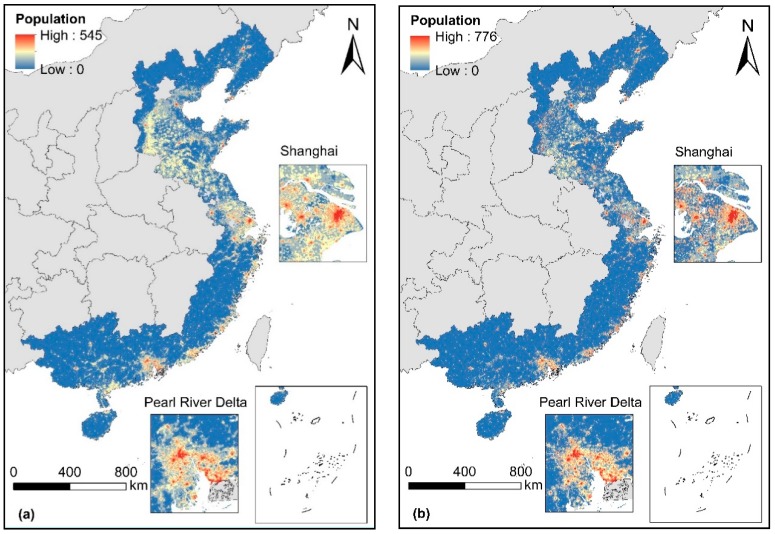
Predicted population density map by (**a**) RF and (**b**) Cubist models for 2010 in coastal China.

According to statistical results (Section 3.1), the performance of the RF model was slightly better than that of the Cubist model. However, machine learning method is known as the “black box” [27], that is, the same input values produce the same output, and the model itself does not explain the actual world. The performance should be further examined by analyzing the spatial distribution of the predictions from the RF and cubist models. Two predictions showed similar spatial patterns, and high-density population was concentrated in urban areas, especially in the Yangtze River Delta and the Pearl River Delta (Figure 4).

**Figure 5:**
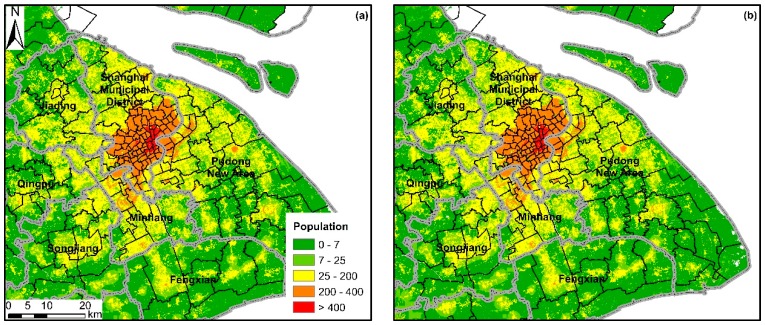
Comparison of population distribution predicted by (**a**) RF and (**b**) Cubist models in downtown Shanghai. Gray lines denote the boundary at county level, and black lines denote the boundary at Jiedao/Xiangzhen level.

We further compared the difference between the two predictions of the RF and Cubist models in the urban area of Shanghai (Figure 5). The spatial distribution of the prediction from the RF model was sprawl, whereas that from the Cubist model exhibited aggregation in urban centers. This result can be related to the characteristics of the two machine learning methods. The predicted value of the RF model was limited to the range of the dependent variable used for modeling, whereas that of the Cubist model can be extrapolated appropriately. We used the natural logarithm of population density at the county level as the dependent variable, with the values ranging from 1.33 to 8.25. As a result, the pixel values of weight layers predicted by the RF and Cubist models ranged from 1.54 to 8.06 and 0.07 to 8.79, respectively. This phenomenon also explained why the population distribution predicted by RF was even, and that by Cubist was concentrated. This result indicated that the RF model reflects the actual population distribution in the coastal provinces of China.

### 3.3. Variable Importance in Population Mapping

**Figure 6:**
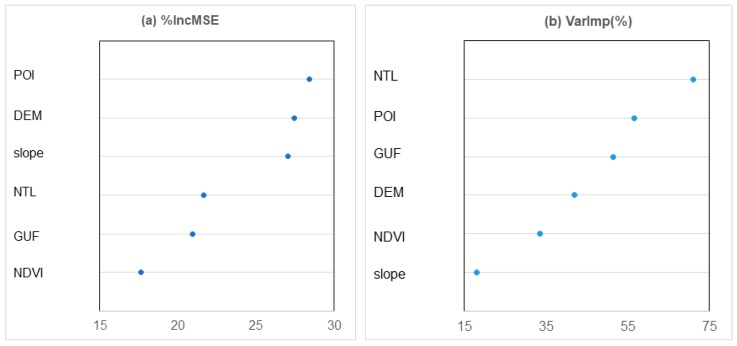
Variable importance for (**a**) RF and (**b**) Cubist regression models.

Given the six covariates used for population estimation, we expect to determine which of these drivers and covariates are the most important in terms of their ability to represent the distribution of population. Figure 6 shows the importance of the covariates in the RF and Cubist models. We chose %IncMSE as the measure of variable importance in the RF model. The %IncMSE indicates the increase in the MSE of prediction (i.e., population in this study) as a result of one variable being permuted. The higher the value of %IncMSE is, the more important the variable is for the regression of the RF model. For the Cubist model, each predictor had a value of the VarImp (%), which is a linear combination of the usage of each variable in the rule conditions and model. We used this value to measure the importance of each predictor in the Cubist model.

According to Figure 6, POI density was the most important predictor in the RF model and the second most important predictor in the Cubist model. Human activities generally take place in different types of POIs. The higher the POI density is, the more developed infrastructures will be and the more service industries there will be. POIs that are highly related to human daily life can better represent an area with high population density and exclude industrial regions than NTL [28]. In contrast, our previous population mapping in China using the RF model showed that slope is the most important predictor [9]. This result indicated that the main geographic variables driving population distribution vary among regions. Variables for machine learning methods should be carefully selected according to the characteristics of the study area and research scale.

Although DMSP/OLS NTL data are widely used to estimate populations across the world, several limitations, such as the blooming effect and saturation, limit the utility of NTL data for accurately estimating population distribution [29,30]. A main problem is that the lit area on DMSP/OLS is much larger than actual urban area due to blooming effect [31,32,33]. Therefore, errors and limitations exist when DMSP/OLS NTL data are used to map urban extents and population distribution. The NTL was much less important than POI density in the RF model, whereas the NTL was the most important indicator in the Cubist model. This phenomenon may explain the higher accuracy of the RF model than the Cubist model.

Elevation and slope are also important indicators in the RF model. This finding should not be a surprise and agreed with expectations that more than 85% of the Chinese population lives in low-relief-degree areas, and the correlation between relief degree and population density over China is strong [34]. Most human settlements are located at low elevation [35,36]. The high-resolution human settlement data also provided detailed information about the presence of population. Vegetation cover is closely and negatively correlated with impervious surfaces [37]. Combining information from NDVI can considerably enhance urban features and improve the mapping population distribution [38]. Therefore, GUF and NDVI also contribute to population prediction.

### 3.4. Contribution of POI Data

Despite numerous efforts to improve and standardize population census procedures, obtaining reliable small-area population estimates faces important challenges in many parts of the world, especially in developing countries. For example, the WorldPop project developed a machine learning-based dasymetric redistribution approach for mapping population at fine spatial resolution that has been shown to improve the accuracies of previous approaches [39]. Nevertheless, the underestimation in high-population-density areas and the overestimation in low-population-density areas, which are frequently recurring problems due to spatial nonstationarity in studies on dasymetric mapping [26,40,41], remain in the WorldPop dataset.

Built area-related covariates are the most important factors in predicting population density [42]. The satellite-derived maps of land cover, NTL, and human settlements are widely used as auxiliary information in the population disaggregation process. Although remote sensing data perform well in discovering physical characteristics, such as land surface reflectivity, texture of urban land, and lit areas that are correlated with population densities, they do not perform well in identifying and understanding social structure and functions of urbanized areas [43,44] and are not directly indicative of the presence of population. Many studies rely on ancillary information obtained from remotely sensed data, but the resolution of the imagery used is often very low to obtain accurate disaggregation results, especially in heterogeneous urban environments. Therefore, a number of experiments demonstrated that land use and NTL data cannot be used to conduct accurate estimation of population at a fine scale [45].

POIs capture human activities better and are more sensitive to socioeconomic environments than remotely sensed data. Baidu’s POI taxonomy consists of 20 top-level category types. As an analogy to spectral signatures in remote sensing, semantic signatures can differentiate types of places [46]. POI is better in representing urban areas, building footprints of residential areas, and rural settlement relative to NTL and land cover data. The inclusion of detailed information on the location and type of residential units can remarkably benefit dasymetric mapping [26,47,48,49]. Thanks to their thematic richness, POIs allow residential and nonresidential uses of urban land to be partly discerned, which is beneficial in disaggregating population. Information extracted from POI and the remote sensing imagery can validate each other to yield precise results in population estimation, especially in urban areas.

**Figure 7:**
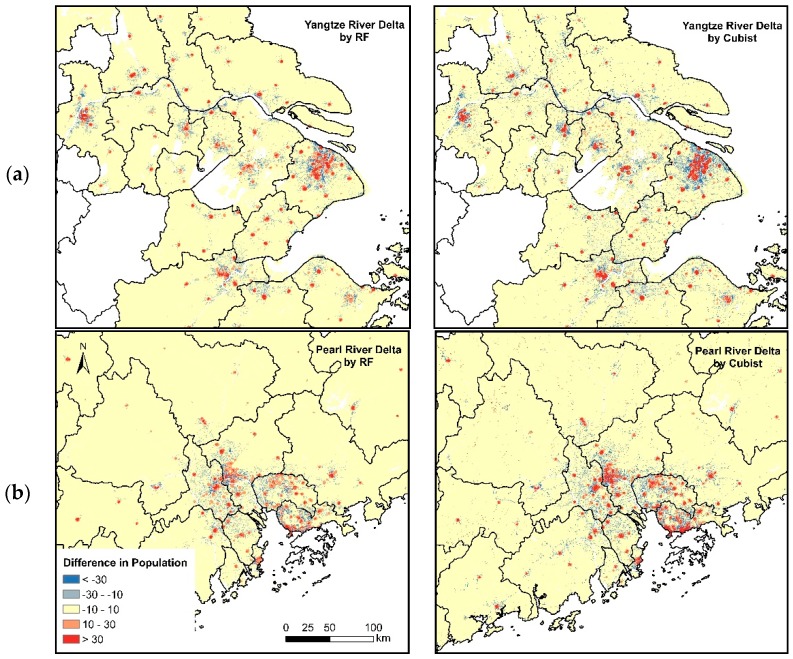
Differences between predictions with and without using POI data in (**a**) Yangtze River Delta and (**b**) Pearl River Delta by subtracting prediction dataset without using POIs from POI-combined dataset.

To evaluate the contribution of POIs data in estimating population distribution, we removed the POI density and used only the remaining five variables, namely, elevation, slope, NDVI, NTL, and GUF, as the independent variables to fit RF and Cubist models and predict population maps. Similarly, we used the census data at the Jiedao/Xiangzhen level to assess the accuracy. The prediction of RF and Cubist models without POI data yielded *R*^2^ values of 0.89 and 0.89, respectively. Compared with the prediction with POI data, the estimation of the population without POI data had large MAE, MRE (%), and RMSE values. The MAE values were 13,742.17 and 15,473.67, the MRE (%) values were 46.66% and 49.06%, and the RMSE values were 23,277.41 and 25,420.24 for RF and Cubist predictions, respectively. Figure 7 shows the differences between predictions in Shanghai and the Pearl River Delta with or without POI data. In addition to producing an accurate model, the predictions incorporating POIs showed that the population was distributed in highly urbanized areas, and population in the suburbs was decreased. The resulting population dataset represented a remarkable improvement in accuracy relative to the WorldPop dataset, which uses remotely sensed and infrastructure-related variables to map population data. The results of this study demonstrated that compared with the mainland China population density map from WorldPop, the inclusion of POI data overcame the problems of underallocation in urban areas and overallocation in rural areas (Figure 7). As a result, the RF and Cubist models incorporating POI data performed much better than the WorldPop model and can successfully predict high population densities in highly urbanized areas in coastal China, such as Shanghai and Guangzhou.

### 3.5. Population Distribution in LECZ

**Figure 8:**
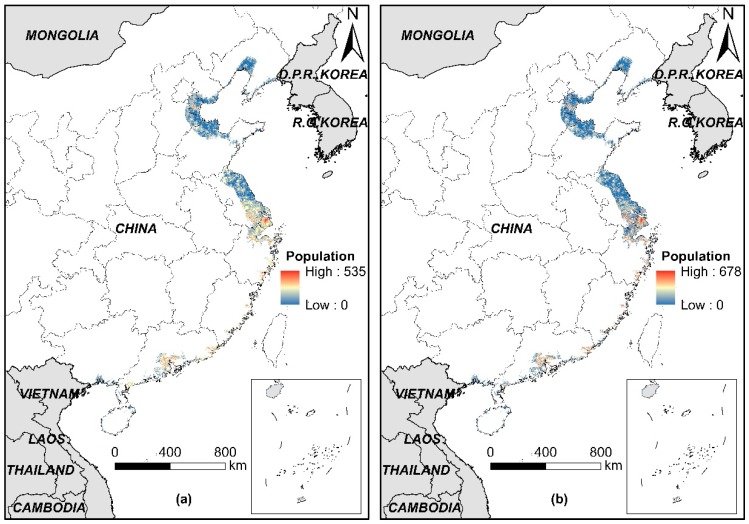
Spatial distribution of population in China’s LECZ on the basis of population data predicted by (**a**) RF and (**b**) Cubist models.

The total numbers of the exposed population in China’s LECZ in 2010 were 158.2 million (RF prediction) and 160.6 million (Cubist prediction) by overlaying the predicted population maps and the extent of LECZ layer. These figures account for 12.17% and 12.36% of the total national population, respectively. Compared with the global average value of 10% [3], an increasing number of residents in China are exposed to coastal lowlands. Liu, Wen [8] analyzed the population distribution in China’s LECZ in 2010 with the GPWv3 population data. The results showed that in 2010, ~163.9 million people lived in China’s LECZ, accounting for 12.3% of China’s total population. This result indicated the credibility of our results. Figure 8 shows that areas with high population density in LECZ were located along the southeast coast, especially in urban areas.

Table 4 shows the exposed population of each province and its proportion to the total population of the province. Compared with our results, the total number of exposed population and the proportion of exposed population in each province on the basis of WorldPop dataset were underestimated. The total exposed population in China’s LECZ according to WorldPop data was 126.2 million, accounting for 9.71% of the country’s total population. According to the RF and Cubist predictions, the differences in the population distribution in LECZ between provinces were significant. Hainan Province had the lowest exposed population of only ~1.6 million, whereas Jiangsu Province and Guangdong Province had the highest population of >34 million people. Shanghai had the largest proportion of ~85.5% of exposed population to the total population, whereas that of Guangxi was the smallest at only ~2.7%. Areas with densely exposed populations were concentrated in Tianjin, Guangdong, and the Yangtze River Delta Economic Zone (including Shanghai, Jiangsu, and Zhejiang), accounting for 78% of the total exposed population. These provinces also had the most developed economies and the highest urbanization levels in the coastal areas of China.

## 4. Limitations

Although the results presented here make a strong case for the integration of POI densities in improving population mapping accuracies, a number of limitations and drawbacks should be addressed. First, most POIs concentrate in urban areas in coastal China, and small cities or rural areas may not have rich data. Therefore, the improvements presented in this study may possibly be limited to developed urban regions. In rural areas and urban fringe areas, where human activity density is relatively low, POI data are an ineffective measure of population density. Second, although POIs can provide the location of socioeconomic activities, POIs cannot provide the extent of these activities. The lack of information on the volume of buildings may generate population underestimation or overestimation [40]. If the extent of a POI can be obtained, then the performance of our method will be further improved. Further research is required to deal with the data availability of building volume or height in large-scale application. Finally, the limited accuracy and resolution of the open-access DEM data lead to uncertainty in population estimation in China’s LECZ, especially in the risk assessment of coastal inundation and erosion [50,51]. In the future, very high resolution DEM data can improve the accuracy of population exposure estimation in China’s LECZ.

## 5. Conclusions

In this study, we used multisource remote sensing images and POI data to disaggregate the census population data in China’s coastal provinces and municipalities by using two machine learning methods, namely, RF and Cubist. The predicted population distribution maps with a spatial resolution of 100 × 100 m were produced in China’s coastal areas in 2010. Our predictions were more accurate and can better capture the characteristics of actual population distribution than the WorldPop dataset. The inclusion of the POI data overcame the problem of population underallocation in urban areas and overallocation in rural areas in coastal cities. This study shows the potential of POIs data to assist in estimating other socioeconomic factors in the future.

Our results showed that <12% of the Chinese population were located in LECZ in 2010. The exposed population were underestimated based on the WorldPop dataset. These estimates are basic but critical information in developing sustainable adaptation strategies to reduce coastal vulnerability to climate change [52]. Against the background of growing risks and rapid urbanization in China’s LECZ, effective, immediate, and long-term adaptation strategies are needed in reducing risks in the coastal systems and low-lying areas.

## Figures and Tables

**Table 1:** Datasets used in this study.

Dataset	Format	Source
POIs (2010)	Point features	Baidu Map Services (http://map.baidu.com)
Nighttime light (2010)	Grid	The National Oceanic and Atmospheric Administration’s National Geophysical Data Center (NGDC), USA (https://ngdc.noaa.gov/eog/dmsp/download_radcal.html)
NDVI (2010)	Grid	Vlaamse Instelling Voor Technologish Onderzoek, Belgium (http://www.vgt.vito.be/)
GDEM	Grid	The Earth Remote Sensing Data Analysis Center (ERSDAC), Japan (http://www.gdem.aster.ersdac.or.jp/search.jsp)
Census population data (2010)	Table	National Bureau of Statistics of China
Global Urban Footprint (GUF) (2011–2012)	Grid	German Aerospace Center (DLR), (https://www.dlr.de/eoc/en/desktopdefault.aspx/tabid-9628/16557_read-40454/)
Boundary maps	Polygon features	Administration of Surveying Mapping and Geoinformation, China
WorldPop Mainland China dataset (2010)	Grid	WorldPop China Mainland dataset: people per pixel (‘ppp’) (http://esa.un.org/wpp/)

**Table 2:** POI categories.

Category	Counts	Category	Counts
Governmental agency	192,196	Commercial Building	20,465
Airport	311	Retail	591,372
Railway station	666	Hotel	71,622
Motorcycle station	3729	Restaurant and entertainment	380,637
Bus station	145,049	Hospital and clinic	78,867
Gas station	39,583	Educational facility	134,506
Factory	81,018	Company	492,264
Service zone of highway	10,873	Parking lot	75,415
Toll station	6461	Residential community	91,065
Bank	154,266	Park and square	7159

**Table 3:** Overall accuracy evaluation for RF and Cubist models results and WorldPop datasets using census data from 13,065 Jiedao/Xiangzhen.

	RF	Cubist	WorldPop
Mean	42833.51	42830.36	44363.85
MRE (%)	41.73	39.87	56.01
MAE	11809.11	11898.52	15996.73
RMSE	19999.14	20270.09	28190.86
%RMSE	46.54	47.16	65.60

**Table 4:** Population statistics in China’s LECZ at province level.

Administrative Region	RF		Cubist		WorldPop	
	Population in LECZ	Percentage of Population (%)	Population in LECZ	Percentage of Population (%)	Population in LECZ	Percentage of Population (%)
Liaoning	5,744,143	13.52	5,865,836	13.80	4,348,797	10.23
Hebei	7,544,156	10.77	7,532,371	10.75	5,959,338	8.51
Tianjin	8,626,425	78.62	8,676,403	79.07	8,050,853	73.37
Shandong	10,543,409	11.34	10,473,863	11.27	8,301,668	8.93
Jiangsu	34,691,242	47.11	34,544,677	46.91	29,578,425	40.17
Zhejiang	26,552,658	49.25	27,594,627	51.18	19,506,037	36.18
Shanghai	19,383,507	85.11	19,586,423	85.99	15,432,892	67.76
Fujian	7,794,071	21.57	8,404,211	23.26	5,624,798	15.57
Guangdong	34,549,207	33.51	34,973,304	33.92	26,907,720	26.10
Guangxi	1,190,414	2.62	1,262,995	2.78	1,070,519	2.36
Hainan	1,589,500	20.42	1,724,000	22.14	1,428,907	18.35
